# Optimal Cut-Off Values for Body Mass Index and Fat Mass Index Based on Age in Physically Active Males Using Receiver Operating Characteristic Curve

**DOI:** 10.3390/diseases11040137

**Published:** 2023-10-06

**Authors:** Daisy Masih, Gurseen Rakhra, Annu Vats, Saroj Kumar Verma, Vijay Kumar Singh, Vandana Kirar, Jitendra Kumar Tripathi, Som Nath Singh

**Affiliations:** Nutrition Division, Defence Institute of Physiology and Allied Sciences, (DIPAS), Delhi 110054, India; gurseen.bhatia@gmail.com (G.R.); annuvats.nona@gmail.com (A.V.); sirfsaroj@gmail.com (S.K.V.); singhvijay75@gmail.com (V.K.S.); vandanadrdo@gmail.com (V.K.); nutrition-dipas@hotmail.com (S.N.S.)

**Keywords:** bioelectric impedance analysis, fat mass, obesity, overweight, Youden index

## Abstract

This study aims to redefine obesity cut-off points for body mass index (BMI) and fat mass index (FMI) according to the different age groups of physically active males. Healthy physically active volunteers (N = 1442) aged 18–57 years (y), with a mean BMI = 22.7 ± 2.8 kg/m^2^, and mean FMI = 4.3 ± 1.7 kg/m^2^ were recruited from various fitness centers. BMI was calculated and individuals were categorized according to the Asia–Pacific BMI criterion of ≤22.9 kg/m^2^ and the previous WHO-guided BMI criterion of ≤24.9 kg/m^2^. FMI was also calculated for the study participants with a cut-off of 6.6 kg/m^2^. Redefining of BMI and FMI cut-off values was carried out based on different age groups categorized with a difference of 10 y and 5 y using the receiver operating characteristic (ROC) curve and Youden’s index. For the entire study population, BMI redefined cut-off points for overweight and obesity were 23.7 kg/m^2^ and 24.5 kg/m^2^, respectively, while FMI redefined cut-off points for overweight and obesity were 4.6 kg/m^2^ and 5.7 kg/m^2^, respectively. With 10 y of age group difference, a constant BMI and FMI values were observed, while with 5 y of age group difference, a constant increase in the BMI cut-offs was observed as the age group increased, i.e., from 23.3 kg/m^2^ in 20–24 y to 26.6 kg/m^2^ in ≥45 y and a similar trend was seen in FMI cut-offs. To conclude, our study suggests that age-dependent BMI and FMI cut-off points may provide appropriate measurements for physically active males as the age group increases.

## 1. Introduction

BMI is easy to calculate, used as a metric for overall mortality prediction, as well as a substitute for body composition, and is the recommended screening tool for measuring obesity in large populations [1]. The World Health Organization (WHO) retained BMI cut-off point 18.5–24.9 kg/m^2^ (normal range) as international classification and endorsed BMI cut-off point 18.5–23.0 kg/m^2^ (normal range) for Asia–Pacific populations for assessing healthy body weight [2]. However, BMI is not considered an appropriate gold standard for the measurement of obesity since it is based on total body weight and does not distinguish between body fat mass and lean body mass [3].

The cut-off points for BMI are based on the results of the meta-analysis involving mainly results from nine countries in Asia as per the WHO expert consultation report [2]. The main concern for Asian populations was the increased risk of diseases like diabetes, heart disease or other chronic diseases, and mortality at lower BMIs [2]. Thereby, limiting the other factors such as age, gender, physical activity, living style, and somatotypes as secondary concerns. However, all these secondary concerns are quite influential while calculating BMI, e.g., women have approximately 10% higher body fat compared to men [4]. 

Another measure to predict adiposity is fat mass index (FMI) which is calculated by replacing total body weight with fat mass in the BMI formula. This gives a fat mass index (FMI) which provides the advantage of determining obesity only in one component, i.e., fat mass while giving a more specific prediction [5]. FMI is expressed as kg/m^2^ while represented by the formula FMI = FM (kg)/height (m^2^). Rao et al., 2012 proposed cut-off points for FMI as 6.6 kg/m^2^ for the Indian population with a body fat percentage of 25% for men and 30% for women to determine obesity [5]. Body fat percentage and FMI are also known to be a good screening tool for metabolic syndrome [6].

Physical activity helps to reduce fat mass and maintain lean body mass. Physically active individuals have additional lean body mass with a disproportionate lesser increase in body fat [1]. This additional lean body mass results in an overestimation of obesity in physically active individuals such as bodybuilders, athletes, and armed personnel, even though their health status is not directly associated with diseases related to excess body fat [1,7,8]. BMI, due to the inability to discriminate between body fat mass and lean body weight, generally misclassifies muscle mass as body fat in physically active individuals [9]. Though minor, the prevalence of physically active individuals with this additional weight exists around us. Studies conducted on athletes and non-athletic groups reported that only 52.4% of the physically active population was correctly classified using BMI [10]. A study reported cut-off points of BMI = 24.38 kg/m^2^ and FMI = 3.74 kg/m^2^ among physically active men to predict the risk of musculoskeletal injury. This physically fit and active adult population has not yet been sufficiently given importance to optimize cut-off points for any obesity measuring indices [11]. Since adiposity increases with age, age also plays a vital role in determining the obesity measuring indices. It was recently published that BMI increases with the service duration of the German Armed Forces, highlighting the fact that BMI increases with age [12].

Since the criterion is important to address any physically active individual as healthy or unhealthy, here we compared the previously accepted and recommended cut-off methods by WHO and tried to provide a glance at the effect of age on BMI and FMI in physically active individuals. BMI and FMI were studied in different age groups of physically active male volunteers using the body fat percentage cut-offs of 20% and 25% to define overweight and obesity, respectively. There are multiple studies published reporting varying cut-offs for entire study groups [13,14,15]; however, only a few are published with different age groups and require more studies to evaluate age–BMI trends as suggested also by Silveira EA et al., 2020 [16,17]. Additionally, this study further redefines cut-off values for BMI and FMI for a population who are physically fit and active using the receiver operating characteristic (ROC) curve.

This study aims to redefine obesity cut-off points for BMI and FMI according to the different age groups of physically active males using the ROC curve.

## 2. Materials and Methods

### 2.1. Participants

The cross-sectional study design was used for the recruitment of healthy participants (N = 1442) from various fitness centers located in Delhi and NCR (National Capital Region). Amateur healthy volunteers aged 18 to 60 years (y), with a mean time of fitness exercise of at least 2 h/day or 12–14 h/week and who gave written consent were included in the study. These participants were doing fitness exercises experience ranging from 2 months to 120 months. Participants were on a balanced diet with adequate carbohydrates, proteins, and fats. Dietary intake was assessed on limited participants using a self-filled food diary for 7 days, which included all the consumed drinks and food items as the participants were free living. The energy intake was calculated using the database of National Institute of Nutrition (http://218.248.6.43:8080/CountWhatYouEat/, accessed on 1 July 2020) and dietary manual (https://www.nin.res.in/downloads/DietaryGuidelinesforNINwebsite.pdf, accessed on 1 July 2020 [18]), though the method has its own limitations of over- and underreporting food intake [19,20]. Male participants (n = 24) aged <18 y with a prolonged or newly diagnosed history of medication due to acute or chronic illness were excluded from the study. Also, due to a limited number of female participants (n = 24), the data collected from them was not statistically sufficient to draw significant results and, thus, they were also excluded from the study.

Out of the 1442 participants, only 1394 were included in the study with an average age of 29 (18–57) y, weight—66.8 (44.5–100.8) kg and height—171.4 (154–187.1) cm. The sample size was calculated using data published in our previous study on different activity groups [21]. We used a formula-based sample size method for sample calculation which was (2 * (1.96 + 0.8416)^2^ * (standard deviation)^2^) divided by (difference in mean of the 2 groups)^2^ [22]. The study was conducted in accordance with the Declaration of Helsinki and approved by the Institutional Ethics Committee of the Defence Institute of Physiology and Allied Sciences (IEC DIPAS, IEC/DIPAS/C-1/2 DATED 26.5.15). Written and informed verbal consent was obtained from all the study participants after explaining to them the details of the study.

### 2.2. Categorization of the Participants 

**Figure 1:**
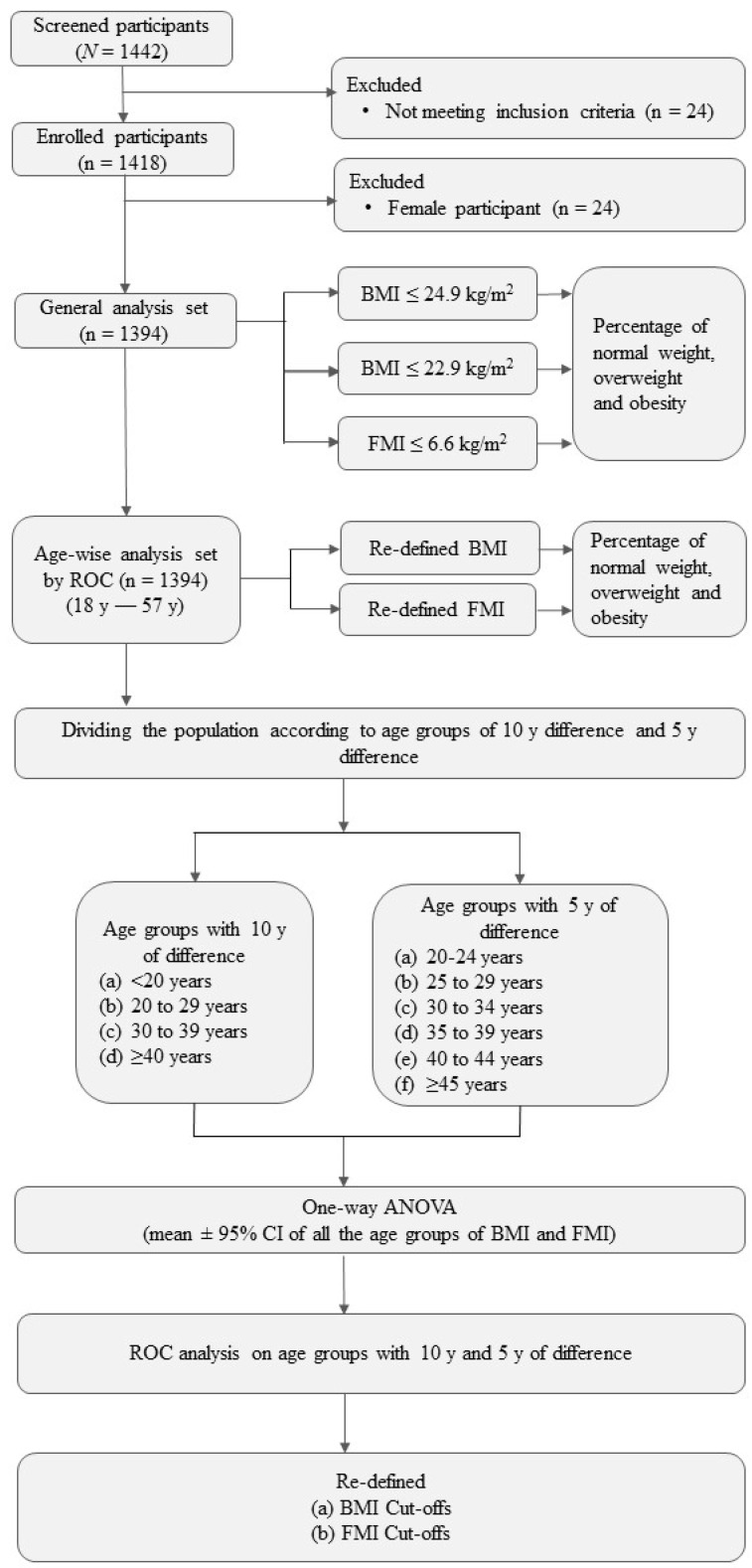
Study design. ANOVA: Analysis of variance; BMI: Body Mass Index; FMI: Fat Mass Index; N: Total Population; n: Populations in the groups; ROC: Receivers Operating Characteristic.

Volunteers were divided based on BMI cut-off points provided by WHO. The cut-off points used were BMI ≤ 24.9 kg/m^2^ and BMI ≤ 22.9 kg/m^2^ according to the international classification as well as revised guidelines for the Asia–Pacific population by WHO, respectively [2]. The subjects were divided into groups based on the international classification as underweight (<18.5 kg/m^2^), normal weight (18.5–24.9 kg/m^2^), overweight (25.0–29.9 kg/m^2^) and obese (≥30 kg/m^2^). The subjects were further re-categorized based on revised consensus guidelines for the Asia–Pacific as underweight (<18.5 kg/m^2^), normal weight (18.5–22.9 kg/m^2^), overweight (23.0–24.9 kg/m^2^), pre-obese (25–29.9 kg/m^2^) and obese (≥30 kg/m^2^). Fat mass index (FMI) was also calculated, and the subjects were categorized as obese if the FMI > 6.6 kg/m^2^ [9]. For redefining the cut-off points, the ROC curve analysis was conducted on the entire population using 20% and 25% percent body fat as standard limits for overweight and obesity, respectively. Further, the population was divided based on 10 y of age groups such as <20 y, 20 to 29 y, 30 to 39 y, and ≥40 y and 5 y of age groups such as 20 to 24 y, 25 to 29 y, 30 to 34 y, 35 to 39 y, 40 to 44 y, and ≥45 y. (Figure 1).

### 2.3. Assessment of Body Composition

#### 2.3.1. Anthropometric and Body Composition Measurements

Height was measured using a measuring rod with the least count of 0.1 cm (Seca 216, Seca Asia Pacific medical measuring systems and scales, Kuala Lumpur, Malaysia). Body weight was measured using a body composition analyzer minimum of 0.1 kg. Body mass index (BMI) was calculated using a pre-defined formula, i.e., weight in kilograms divided by the square of height in meters (kg/m^2^). The day before the body composition measurements, the study was explained to the participants and they were formally informed to fast and refrain from caffeine intake until the measurements were conducted. The body water levels naturally fluctuate throughout the day and night; therefore, the body composition analysis was performed using a bioelectric impedance analyzer (BIA) before breakfast in the morning between 700 h and 1000 h in a post-absorptive state. 

#### 2.3.2. Bioelectric Impedance Analyzer (BIA)

Single-frequency four-electrode BIA (Tanita BC-420MA, body composition analyzer, Tanita Corporation, Tokyo, Japan) was used to measure body composition. The participants were wearing light clothes and removed the belt, rings, and any other metallic items from their bodies before they stood on the flat base of the BIA with bare feet. Fat mass (FM), fat mass percentage (%FM), fat-free mass (FFM), muscle mass, total body water, basal metabolic rate (BMR), and degree of obesity were obtained by regression algorithms as fed by the manufacturer of BIA. BIA measurements were previously validated using dual-energy X-ray absorptiometry (DXA) in a physically active healthy male population [23].

#### 2.3.3. Physical Activity Level (PAL) Value Assessment

Basal metabolic rate (BMR) is the minimum amount of energy the body needs to function effectively when at rest. A multiple of BMR is total energy expenditure (TEE), expressed to determine the requirements of adults. These multiples of BMR are referred to as physical activity levels (PALs) and are assessed by dividing TEE by BMR, i.e., (PAL = TEE/BMR) [24,25]. PAL value delivers a suitable way of controlling for age, sex, weight, and body composition and for expressing the energy needs of a wide range of people in a single number [21]. TEE was measured using accelerometry-based actical devices (Mini Mitter Co. Inc. Bend OR, USA) while BMR was obtained from the BIA measurements. Based on PAL values, the participants can be addressed as sedentary, moderately active, and highly active with PAL values of 1.53, 1.80, and 2.30, respectively [19]. However, the value of PAL depends on both BMR and TEE, and both have errors of measurement, so PAL is only imprecisely estimated [24].

### 2.4. Statistical Analysis

Results were expressed as mean ± standard deviation (SD) along with minimum (min), maximum (max), and interquartile range (IQR) of all the variables (Table 1). The percentages of the prevalence of underweight, normal weight, overweight, and obese were calculated manually. All the statistical analyses were performed using IBM SPSS Statistical Package (version 20.0; SPSS, Chicago, IL, USA), and *p* values ≤ 0.05 were considered significant. One-way ANOVA on mean ± 95%CI of all the age groups of BMI and FMI was calculated along with Bonferroni’s Multiple Comparison Test to investigate the difference significance between the age groups based on BMI and FMI. The receiver operating characteristic curve (ROC curve) was plotted to identify appropriate cut-off points of the BMI and FMI for defining overweight and obesity in the whole study population as well as in different age groups.

The area under the ROC curve (AUC) was used to determine the precision of BMI and FMI that describes the strength of analytical tests. AUC measures sensitivity and specificity which describe the inherent strength of diagnostic tests [26]. Higher AUC values indicate a better model for diagnosis. The diagnostic test with AUC = 1 fits to differentiate between the diseased and non-diseased. The diagnostic test with AUC = 0.5 means the diagnostic test is no better than a chance as the curve is located on a diagonal line in ROC space. The minimum AUC that can be considered a chance level is AUC = 0.5, while the diagnostic test with AUC = 0 is not a suitable diagnostic test as it classifies a diseased state as negative and a non-diseased state as positive [26]. Thus, AUC > 0.5 was considered to have the accuracy to differentiate between a healthy state and overweight/obese state while 95% confidence intervals were recorded for the ROC curve analyses. Body fat percentage was used as the reference standard for diagnosis tests to find out the optimal cut-off point. A Youden index maximum point was used to predict the optimal cut-off points for BMI and FMI. Youden index (J) is the maximum probable cut-off value calculated using the uppermost y-axis distance of the ROC curve from the diagonal line which gives the probability of 50%. The formula used for the Youden Index = Sensitivity + Specificity − 1 [26].

## 3. Results

A total of 1394 healthy physically active male participants with an average age of 29 years (range 18–57 years) were studied for the prevalence of overweight and obesity, along with redefining BMI and FMI cut-off values with increasing age, using receivers operating characteristics (ROC) curve. Both BMI and FMI data obtained were generally distributed in this study. The participants were on an adequate diet with a basal metabolic rate (BMR) of 1600 ± 143 kcal/day and a PAL value of about 2.2, i.e., the participants were moderately physically active. Since we did not have enough actical devices to provide to all the volunteers, we measured the TEE of the few participants (n = 120) whose data was previously published by us [27]. The average TEE of the participants was 3515 ± 915 kcal/day [27]. In addition, the effect of physical activity and age on mineral status in physically active healthy males (n = 360) had already been studied and published by us, which gave us an estimate that the study participants were on adequate diets and were physically active [21]. The physical characteristics and basic body composition parameters of the study participants are depicted in Table 1.

### 3.1. Prevalence of Overweight and Obesity in the Study Population

#### 3.1.1. Body Mass Index (BMI)

With the evaluation of the whole data based on different criteria, 20.7% (n = 289) and 0.9% (n = 12) of the study population were overweight (BMI 25.0–29.9 kg/m^2^) and obese (BMI ≥ 30 kg/m^2^), respectively, with the previous BMI cut-off, i.e., BMI ≥ 24.9 kg/m^2^, while with WHO-recommended BMI criteria for Asia–Pacific populations (i.e., BMI cut-off ≥ 22.9 kg/m^2^), 22.6% (n = 315) of individuals were overweight and 21.6% (n = 301) were pre-obese (BMI 23.0–29.9 kg/m^2^) and obese (≥30 kg/m^2^), in a total of 1394 individuals.

It is to be considered that WHO recommended a new category of pre-obese in the new Asia–Pacific criterion, which comprises individuals whose BMI is between 25.0 kg/m^2^ and 29.9 kg/m^2^. According to this pre-obese condition, 20.7% (n = 289) of individuals fall under this category, making 0.9% of individuals obese (BMI ≥ 30 kg/m^2^). These results illustrate that approximately half of the study population is unhealthy (overweight, pre-obese, and obese) and needs to lower their body weight to become healthy. A statistical analysis of the study population showing the prevalence of physical status in terms of underweight, overweight, and obesity using a different cut-off of BMI and FMI is presented in Table 2.

#### 3.1.2. Fat Mass Index (FMI)

FMI measures obesity by considering only the fat mass of the individuals. FMI divides the population into two categories, i.e., normal weight and obese. Based on this criterion, we observed 91.6% of individuals as normal weight and 8.4% as obese, including overweight individuals. FMI was used to determine the prevalence of physical status which presented a lower prevalence of overweight and obesity with 8.4% (n = 117) of individuals (Table 2).

### 3.2. Evaluating the Study Population Based on BMI and FMI Cut-Off by ROC Curve 

**Figure 2:**
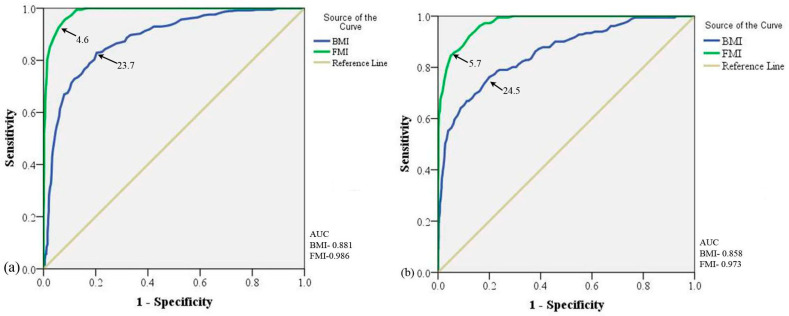
Receiver operating characteristic curve analysis for BMI and FMI in entire population: (**a**) ROC curve for Overweight and (**b**) ROC curve for Obesity. AUC: Area Under Curve; BMI: Body Mass Index; FMI: Fat Mass Index; N: Total Population; n: Populations in the Groups; ROC: Receivers Operating Characteristic. Black arrows pointing BMI and FMI lines.

Since BMI overestimates obesity in physically active individuals, we planned to redefine the cut-off limits for our study population using ROC curve analysis. The cut-off for BMI and FMI for overweight and obesity was best predicted using percent body fat as the standard reference in the ROC curve. The standard body fat percentage used for overweight and obesity was 20% and 25%, respectively. The ROC curve analysis of the entire sample (n = 1394) for overweight and obesity cut-off yielded significant (*p* < 0.0001) higher FMI AUC (95%CI) (Overweight: 0.99 (98.1–99.1%), Obesity: 0.97 (96.5–98.2%)) than BMI AUC (95%CI) (Overweight: 0.88 (86.1–90.2%), Obesity: 0.86 (82.6–88.9%)); (*p* < 0.0001) thereby, indicating FMI as a better predictor for the obesity index (Figure 2). The BMI cut-off point for overweight was 23.7 kg/m^2^ (Sensitivity: 71.3%, Specificity: 89.3%) and for obesity, it was 24.5 kg/m^2^ (Sensitivity: 76.2%, Specificity: 80.0%). The FMI cut-off point for overweight was 4.6 kg/m^2^ (Sensitivity: 95.7%, Specificity: 91.8%) and for obesity, it was 5.7 kg/m^2^ (Sensitivity: 92.3%, Specificity: 87.7%) (Figure 2). The prevalence of nutritional status of the population by ROC curve produced BMI and FMI cut-off defined by the present study is compared to the previous and new WHO-recommended BMI cut-offs presented in Table 2.

### 3.3. Defining Cut-Offs Based on Different Age Groups 

**Figure 3:**
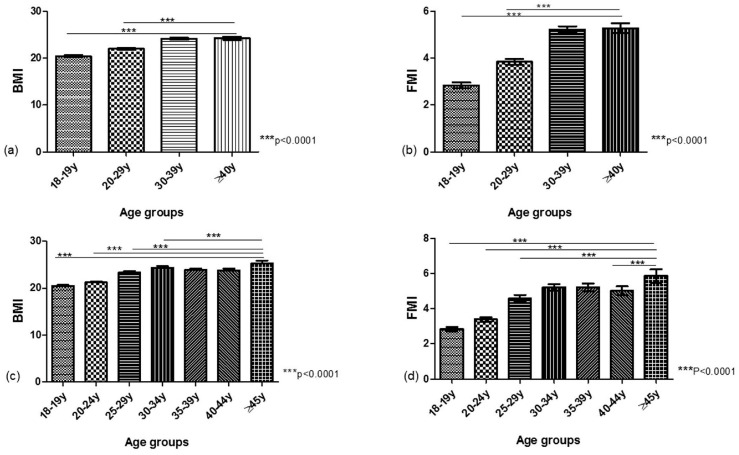
Comparison of BMI (Mean ± 95%CI) and FMI (Mean ± 95%CI) within age groups using one-way ANOVA: (**a**) BMI with difference of 10 y of age; (**b**) FMI with difference of 10 y of age; (**c**) BMI with difference of 5 y of age; (**d**) FMI with difference of 5 y of age. ANOVA: Analysis of Variance; BMI: Body Mass Index; FMI: Fat Mass Index; y: Years.

Before continuing with ROC on different age groups, we applied one-way ANOVA on a mean ± 95%CI of all the age groups of BMI and FMI along with Bonferroni’s Multiple Comparison Test. This resulted in a significant (*p* < 0.0001) increase in BMI and FMI with increasing 10 y of age, except for 30–39 y vs. ≥40 y, which were not significant. The age groups with 5 y of difference showed significant (*p* < 0.0001) increases in BMI and FMI with increasing age, except for some BMI and FMI groups, which were not significant. Figure 3 shows a comparison of BMI (Mean ± 95%CI) and FMI (Mean ± 95%CI) within age groups using one-way ANOVA: (a) BMI with a difference of 10 y of age; (b) FMI with a difference of 10 y of age; (c) BMI with a difference of 5 y of age; (d) FMI with a difference of 5 y of age. Table 3 shows one-way ANOVA Bonferroni’s Multiple Comparison Test showing the significance of the difference between various age groups in BMI and FMI.

**Figure 4:**
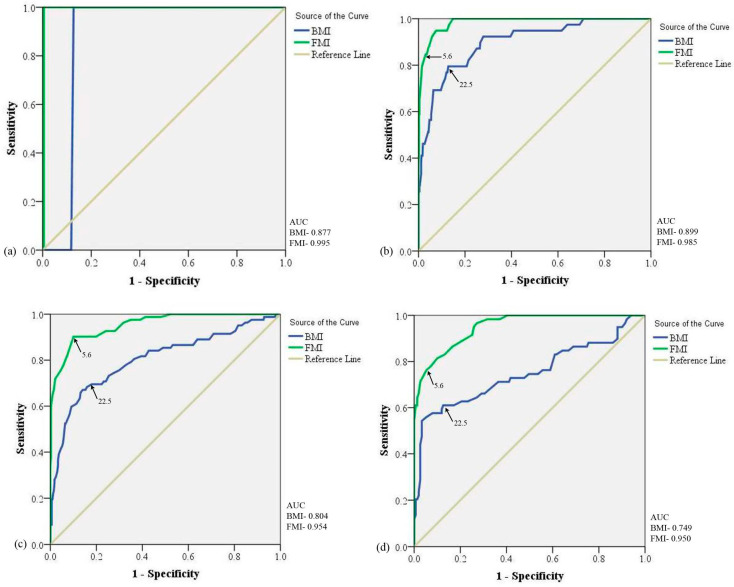
Receiver operating characteristic curve analysis for BMI and FMI with 10 years of difference in age groups: (**a**) age group below 20 years; (**b**) age group 20 to 29 years; (**c**) age group 30 to 39 years; and (**d**) age group ≥40 years. AUC: Area Under Curve; BMI: Body Mass Index; FMI: Fat Mass Index.

**Figure 5:**
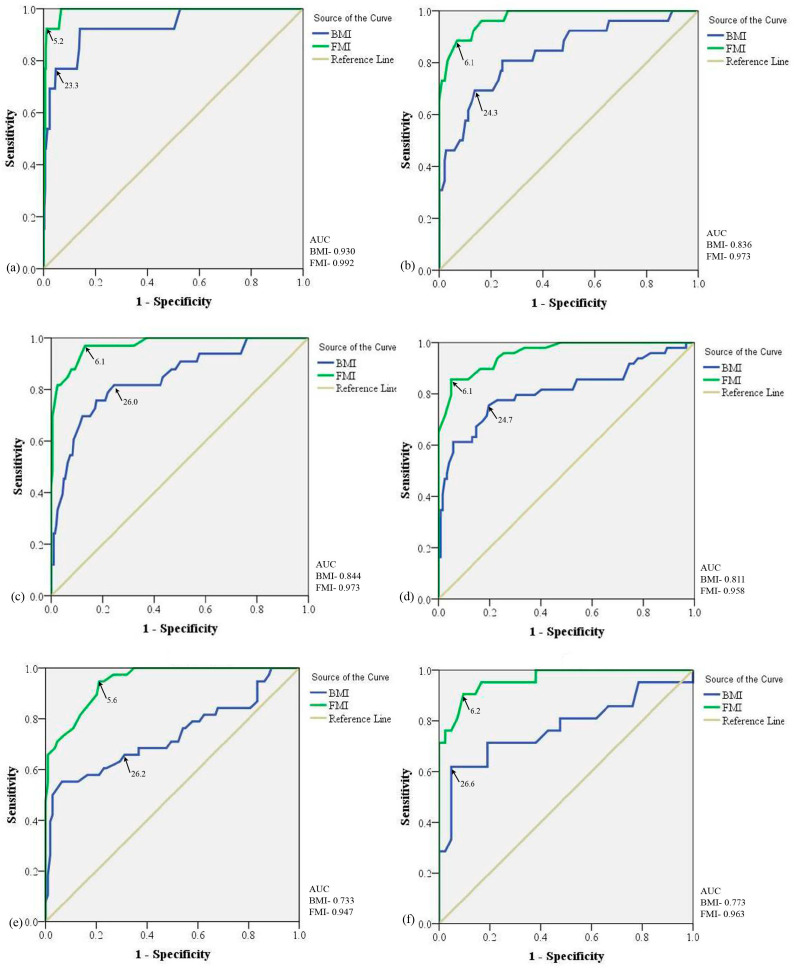
Receiver operating characteristic curve analysis for BMI and FMI with 5 years of difference in age groups: (**a**) age group 20–24 years; (**b**) age group 25 to 29 years; (**c**) age group 30 to 34 years; (**d**) age group 35 to 39 years; (**e**) age group 40 to 44 years; and (**f**) ≥45 years. AUC: Area Under Curve; BMI: Body Mass Index; FMI: Fat Mass Index.

To find the BMI and FMI trends with increasing age, grouping of the individuals was carried out based on an age difference of 10 years (Figure 4) and 5 years (Figure 5) for both indices. 

#### 3.3.1. Age Group with 10 Years of Difference

We divided the population based on 10 y of age group differences such as <20 y, 20 to 29 y, 30 to 39 y, and ≥40 y. Using ROC curve analysis, we observed the cut-off of BMI (22.5 kg/m^2^) for all age groups with similar sensitivity and specificity which were 100% and 87.3%, respectively. The cut-off of FMI (5.6 kg/m^2^) was also the same for all age groups with similar sensitivity and specificity which were 100% and 99.5%. Table 4 specifies the area under the ROC curve with a 95% confidence interval of the different age groups depicting cut-off values for obesity with %BF ≥25%. It can be noticed here that on the division of the study population based on 10 y, the cut-off limits were reduced by 2.0 kg/m^2^ and 0.05 kg/m^2^ for BMI and FMI, respectively.

#### 3.3.2. Age Group with 5 Years of Difference

We divided the population based on 5 y of age group differences such as 20–24 y, 25–29 y, 30–34, 35–39 y, 40–44 y and ≥45 y. In ROC curve analysis, we observed a constant increase in the BMI cut-offs as the age group increases, i.e., from 23.3 kg/m^2^ in the age group 20–24 y to 26.6 kg/m^2^ in the age group ≥45 y. Table 4 specifies the area under the ROC curve with a 95% confidence interval of the different age groups depicting cut-off values for obesity with %BF ≥25%. To our surprise, a drop of 1.3 kg/m^2^ in BMI cut-offs was seen in the age group 35–39 y (Figure 5d). An almost similar increasing trend was observed in FMI cut-offs as the age groups increased as in BMI cut-offs with a drop of 0.5 kg/m^2^ in the age group of 40–44 y. Diversity in the obesity prevalence is noted with the diversification of ROC-analyzed BMI and FMI cut-offs. According to re-defined FMI cut-offs, an increase in obesity percent was depicted with an increase in age group. However, no such pattern was seen to be associated with BMI. Table 5 represents the cut-off points of BMI and FMI for the different age groups with sensitivity and specificity analyzed by the ROC curve for obesity with %BF ≥25%.

## 4. Discussion

This study aimed to redefine cut-offs of BMI and FMI for physically active individuals in relation to the increasing age. In this study, the prevalence of overweight and obesity was observed as 22.6% and 21.6%, respectively, with the WHO-recommended Asia–Pacific BMI cut-off point, which is 22.9 kg/m^2^. The prevalence of obesity in the study population is assumed to be very high in comparison to the National Family Health Survey 5 (2019–2021) of India where 22.9% of men were reported as overweight and obese in the country (considering previously defined BMI cut-off was ≥25.0 kg/m^2^) [28]. This difference in the prevalence of obesity might be due to the application of the new Asia–Pacific BMI cut-off point. As per WHO, the requirement for population-specific BMI cut-off was essential due to the difference in the body fat and health risks present at similar BMI in Asian countries in comparison to European countries [2]. However, the data used was not sufficient to specify a stringent BMI cut-off point for all Asians for overweight or obesity [2]. Thus, there may be a discrepancy in measurement technique as well as the obesity measuring guidelines, which may lead to increased obesity rates observed in the present study.

Looking at the published literature, many factors affect the body fat distribution in Asian Indians [29]. These factors may include age, gender, lifestyle, physical activity, and genetic risk. It is very well stated that socioeconomic position can affect a person’s health status. A study representing the trends of overweight/obesity in India based on socioeconomic status showed an increased prevalence of 8% from the year 1998 to 2016 in overweight/obesity among Indian literate urban men [30]. The same authors forecasted the prevalence of overweight and obesity in India to increase by 17.9% and 7.1%, respectively, from 2010 to 2040 [31]. Similarly, it is observed that urban residents of India are more prone to obesity than rural residents as also reported by the present study [32,33] with a high prevalence of abdominal obesity in Asian Indians [34].

On the other hand, the suitability of the BMI criterion to assess obesity is the most controversial topic of obesity measurements. A debatable topic across Asian countries as well as the world is the use of BMI with lowered cut-off values as Asians show high body fat for similar BMI [35]. It has been reported that body fat percentage varies due to the ethnicity of the individuals, as depicted in the studies conducted with White, African American, and Asian people [36], specifically Japanese [37], Australian [38], Nigerian, and Jamaican [39] populations. These criteria are based on the evaluation of assorted populations and the paucity of precise cut-off values for the index [40]. The variations in body fat percentage are due to different leg-to-trunk lengths, muscularity, and slenderness [41,42]. Gender, age, region, living habits, and lifestyle are the other factors affecting the criterion [43,44,45]. In addition, the combination of these cut-off values associated with the disease may vary between populations [38]. Thus, keeping in mind the suitability of the BMI criterion, ROC curve analysis was performed to find out the suitable and specific BMI and FMI cut-off for these physically fit individuals. ROC curve for the entire study population (18–57 years) indicated a cut-off value of 24.5 kg/m^2^ for BMI and 5.7 kg/m^2^ for FMI. Comparing these results with other published studies on the Indian population, similar results were observed. Kesavachandran et al. proposed 24.5 kg/m^2^ as the BMI cut-off for Lucknow male residents while Ghosh and Bandyopadhyay proposed a 24.0 kg/m^2^ BMI cut-off in Bengalee males [13,14]. Misra et al., 2003 also suggested a 24.0 kg/m^2^ BMI cut-off for males [15]. The BMI cut-off was proposed to be lowered not only in civilians but also in the Indian Armed Forces. Singh et al. advocated for lowering the cut-off values of BMI for Navy personnel and reported 23.9 kg/m^2^ and 24.4 kg/m^2^ cut-off values for overweight and obesity, respectively [46]. Our study worked parallel to Singh et al.’s, 2008 findings and suggests a 23.7 kg/m^2^ and 24.5 kg/m^2^ cut-off of BMI for overweight and obesity, respectively [46].

Many studies support the lowering of the cut-off of BMI for the entire population; however, to the contrary, our study recommends raising the BMI and FMI cut-off values with increasing age. In our study, after dividing the participants based on 10 years and 5 years of age difference, 5 years of age difference gives us better BMI and FMI cut-off trends according to the increasing age. However, to date researchers have proposed the cut-off values using the entire sample of all the age groups; for example, Misra et al., 2003 and Kesavachandran et al., 2012 both proposed lowering the cut-off values considering all age groups from 18 years to 82 years [14,15]. It should be kept in view that the body fat percentage and age are two discriminating factors, and it can hamper the precision of the results. Also, our study supports FMI as a better predictor of obesity for individuals of all age groups. This finding best shores up with earlier findings by those who encourage FMI over BMI [47,48]. Thus, age-independent BMI might produce inaccurate measurements of health status.

Strengths: This study focused on the health status of physically active individuals who are sometimes considered overweight or obese by BMI measurements due to more lean mass. One of the strengths of this study is that it represents a comprehensive examination of the whole BMI screening criterion where cut-off values were marked according to the age groups. Another strength includes the geographical area of the study population which is Delhi and the National Capital Region as the inhabitants belong to all the parts of the country which makes the results reliable for conclusion.

Limitations: First, this study was observational, and participants were not on a pre-planned structured training or fitness exercise which may have impacted the body composition. Second, the somatotype of the participants was not measured. Third, since females were not included in the study, the cut-off values proposed in the present study should be suggested only for physically active males.

## 5. Conclusions

Physical activity helps to reduce body weight and gain muscle mass. BMI overestimates overweight and obesity leading to the misclassification of muscle mass as body fat in physically active individuals. Adiposity also increases with age; however, limited studies evaluated age-BMI trends. This study re-evaluates cut-off values for BMI and FMI for a population who are physically fit and active using the ROC curve. Our study suggests the possibility of increasing BMI and FMI cut-off values with increasing age in the case of physically active males. The cut-off values for BMI may be considered according to the body fat percentage and age group. Since age-dependent measurements produce better measurements of health status in physically active males; therefore, the criterion may be modified.

## Figures and Tables

**Table 1:** Demographic characteristics of studied participants.

S. No.	Characteristics	Mean	SD	Min–Max	IQR (Q1–Q3)
1.	N	1394	-	-	-
2.	Sex	Male	-	-	-
3.	Age (y)	29	8.7	18–57	14 (21–35)
4.	Height (cm)	171.4	5.0	154–187.1	6.9 (168–174)
5.	Weight (kg)	66.8	8.9	44.5–100.8	13 (60–73)
6.	BMI (kg/m^2^)	22.7	2.8	15.9–35.7	4.0 (20.7–24.7)
7.	FMI (kg/m^2^)	4.3	1.7	1.0–12.3	2.6 (2.9–5.5)
8.	FM (kg)	12.7	4.9	3.1–34.7	7.6 (8.5–16.1)
9.	FM %	18.5	5.3	5.3–35.1	8.7(13.9–22.6)
10.	FFM (kg)	54.5	14.8	30.1–568.5	7.2 (50.5–57.7)
11.	Muscle mass (kg)	53.6	4.7	39.1–73.4	5.5 (50.5–56.0)
12.	Total body water (kg)	38.6	4.2	27.6–56.3	5.4 (35.6–41.0)
13.	Degree of obesity	9.7	11.7	−20.0–62.3	15.1 (2.0–17.1)
14.	BMR (kcal/day)	1600	143	1249–2263	163.3 (1513–1677)

Values are represented as mean ± SD. Min: Minimum; Max: Maximum; IQR: Interquartile Ratio; BMI: Body Mass Index; BMR: Basal Metabolic Rate; FMI: Fat Mass Index; FM: Fat Mass; FM %: Percent Fat Mass; FFM: Fat-free Mass; Q1: Quartile 1; Q3: Quartile 3.

**Table 2:** Comparative prevalence of health status of the study population by WHO recommended previous and new guidelines for Asia–Pacific population along with cut-offs presented by the present study via ROC curve analysis.

Health Status	Previous Cut-Off BMI ≥ 24.9 kg/m^2^	New Cut-Off BMI ≥ 22.9 kg/m^2^	ROC Curve Produced Cut-Off BMI ≥ 23.7 kg/m^2^	FMI ≥ 6.6 kg/m^2^	ROC Curve Produced Cut-Off FMI ≥ 4.6 kg/m^2^
Underweight (cut-off)	4.0% (n = 56) (<18.5 kg/m^2^)	4.0% (n = 56) (<18.5 kg/m^2^)	4.0% (n = 56) (<18.5 kg/m^2^)	-	-
Normal weight (cut-off)	74.3% (n = 1037) (18.5–24.9 kg/m^2^)	51.8% (n = 722) (18.5–22.9 kg/m^2^)	61.3% (n = 854) (18.5–23.7 kg/m^2^)	91.6% (n = 1277) (≤6.6 kg/m^2^)	40.8% (n = 570) (≤4.6 kg/m^2^)
Overweight (cut-off)	20.7% (n = 289) (25.0–29.9 kg/m^2^)	22.6% (n = 315) (23.0–24.9 kg/m^2^)	8.2% (n = 115) (23.8–24.5 kg/m^2^)	-	20.9% (n = 292) (4.6–5.7 kg/m^2^)
Pre-obese (cut-off)	-	20.7% (n = 289) (25.0–29.9 kg/m^2^)	25.6% (n = 357) (24.6–29.9 kg/m^2^)	-	-
Obese (cut-off)	0.9% (n = 12)	0.9% (n = 12) (≥30.0 kg/m^2^)	0.9% (n = 12) (≥30.0 kg/m^2^)	8.4% (n = 117) (>6.6 kg/m^2^)	19.8% (n = 277) (>5.7 kg/m^2^)

Values are represented as percentage (n). BMI: Body Mass Index; FMI: Fat Mass Index; ROC: Receiver Operating Charactristic.

**Table 3:** One-way ANOVA Bonferroni’s Multiple Comparison Test showing significance of difference between various age groups in BMI and FMI.

DIFFERENCE OF 10 Y AGE GROUP							
	**BMI**						
	18–19 y	20–29 y	30–39 y	≥40 y			
18–19 y	-	***	***	***			
20–29 y	***	-	***	***			
30–39 y	***	***	-	ns			
≥40 y	***	***	ns	-			
	**FMI**						
	18–19 y	20–29 y	30–39 y	≥40 y			
18–19 y	-	***	***	***			
20–29 y	***	-	***	***			
30–39 y	***	***	-	ns			
≥40 y	***	***	ns	-			
**DIFFERENCE OF 5 Y AGE GROUP**							
	**BMI**						
	18–19 y	20–24 y	25–29 y	30–34 y	35–39 y	40–44 y	≥45 y
18–19 y	--	**	***	***	***	***	***
20–24 y	**	-	***	***	***	***	***
25–29 y	***	***	-	***	ns	ns	***
30–34 y	***	***	***	-	ns	ns	ns
35–39 y	***	***	ns	ns	-	ns	***
40–44 y	***	***	ns	ns	ns	-	***
≥45 y	***	***	***	ns	***	***	-
	**FMI**						
	18–19 y	20–24 y	25–29 y	30–34 y	35–39 y	40–44 y	≥45 y
18–19 y	-	***	***	***	***	***	***
20–24 y	***	-	***	***	***	***	***
25–29 y	***	***	-	***	***	*	***
30–34 y	***	***	***	-	ns	ns	*
35–39 y	***	***	***	ns	-	ns	***
40–44 y	***	***	*	ns	ns	-	***
≥45 y	***	***	***	*	***	***	-

BMI:Body Mass Index; FMI:Fat Mass Index; y:years; ns:not significant, * *p* < 0.05, ** *p* < 0.001, *** *p* < 0.0001.

**Table 4:** Area under ROC curve with 95% confidence interval of the different age groups depicting cut-off values for obesity with %BF ≥25%.

AGE GROUPS	BMI	FMI
	AUC (95% CI)	AUC (95% CI)
18–60 y (overweight)	0.881 (86.1–90.2%)	0.986 (98.1–99.1%)
18–60 y(Obesity)	0.858 (82.6–88.9%)	0.973 (96.5–98.2%)
**DIFFERENCE OF 10 Y AGE GROUP**		
18–19	0.88 (83.2–92.3%)	0.99 (98.6–100%)
20–29	0.90 (84.7–95.1%)	0.96 (97.4–99.6%)
30–39	0.80 (74.3–86.4%)	0.95 (93.1–97.7%)
≥40	0.75 (66.3–83.4%)	0.95 (92.2–97.7%)
**DIFFERENCE OF 5 Y AGE GROUP**		
20–24	0.93 (85.5–100%)	0.99 (98.1–100%)
25–29	0.84 (74.5–92.7%)	0.97 (94.9–99.8%)
30–34	0.84 (76.7–92.0%)	0.97 (94.9–99.7%)
35–39	0.81 (72.8–89.4%)	0.96 (92.9–98.7%)
40–44	0.73 (62.6–84.0%)	0.95 (91.3–98.1%)
≥45	0.77 (63.2–91.4%)	0.96 (91.9–100%)

BMI—Body Mass Index; FMI—Fat Mass Index; y—years; AUC—Area Under ROC Curve; CI—Confidence Interval.

**Table 5:** BMI and FMI cut-off points for different age groups in the present study population for obesity with %BF ≥25%.

S. No.	Age Groups (y)	Number of Subjects	BMI Cut-Off	Obesity Prevalence (%)	FMI Cut-Off	Obesity Prevalence (%)
1	18–19	205	22.5 (Sensitivity—100%, Specificity—87.3%)	11.7	5.6 (Sensitivity—100%, Specificity—99.5%)	1.0
2	20–24	355	23.3 (Sensitivity—92.3%, Specificity—86.0%)	16.3	5.2 (Sensitivity—100%, Specificity—93.3%)	9.0
3	25–29	215	24.3 (Sensitivity—80.8%, Specificity—75.7%)	30.7	6.1 (Sensitivity—88.5%, Specificity—93.1%)	12.6
4	30–34	238	26.0 (Sensitivity—75.8%, Specificity—82.4%)	24.8	6.1 (Sensitivity—97.0%, Specificity—86.8%)	20.2
5	35–39	171	24.7 (Sensitivity—75.5%, Specificity—80.3%)	33.9	6.1 (Sensitivity—85.7%, Specificity—95.1%)	26.3
6	40–44	147	26.2 (Sensitivity—52.6%, Specificity—95.4%)	17.0	5.6 (Sensitivity—89.5%, Specificity—79.8%)	38.1
7	≥45	63	26.6 (Sensitivity—47.4%, Specificity—97.2%)	23.8	6.2 (Sensitivity—68.4%, Specificity—96.3%)	31.7
8	18–57 (Entire study sample)	1394	24.5 (Sensitivity—76.2%, Specificity—80.0%)	26.5	5.7 (Sensitivity—92.3%, Specificity—87.7%)	19.9

## Data Availability

All data generated or analyzed during this study are included in this published article.
